# Closing the Gap: Advances and Barriers on *Candida* Vaccines Development in the Last Decade

**DOI:** 10.3390/ijms27198490

**Published:** 2026-09-23

**Authors:** Julie Krier, Jontana Allkja, Alexandra Mónica Bastos Viana da Costa, Francisco A. M. Silva, Célia Fortuna Rodrigues

**Affiliations:** 1 Department of Pharmaceutical Sciences, University Institute of Health Sciences (IUCS-CESPU), 4585-116 Gandra, Portugal; a31862@alunos.cespu.pt; 2 Open Innovation Hub for Antimicrobial Surfaces, Surface Science Research Centre, School of Physical Sciences, University of Liverpool, Liverpool L69 3BX, UK; jontana.allkja@liverpool.ac.uk; 3 Associate Laboratory i4HB—Institute for Health and Bioeconomy, University Institute of Health Sciences—CESPU, 4585-116 Gandra, Portugal; alexandra.costa@iucs.cespu.pt; 4 UCIBIO—Applied Molecular Biosciences Unit, Translational Research Laboratory, University Institute of Health Sciences (1H-TOXRUN, IUCS-CESPU), 4585-116 Gandra, Portugal; 5 LEPABE/ALiCE—Laboratory for Process Engineering, Environment, Biotechnology and Energy and Associate Laboratory in Chemical Engineering, Faculty of Engineering, University of Porto, Rua Dr. Roberto Frias, 4200-465 Porto, Portugal

**Keywords:** *Candida* spp., invasive candidiasis, antifungal vaccine, NDV-3A, *Candida auris*, trained immunity, reverse vaccinology, multidrug resistance, RVVC

## Abstract

Invasive Candidiasis (IC) remains a critical threat to global health. Primarily caused by *Candida albicans*, it is increasingly driven by emerging Non-*Candida albicans Candida* Species (NCACs). IC typically arises from the overgrowth of commensal *Candida* species in immunocompromised hosts. IC accounts for an estimated 250,000–700,000 cases annually worldwide, with mortality rates between 40 and 55%, and contributes to more than 1.5 million annual deaths. Despite progress in healthcare systems, intensive care, and microbiological techniques, mortality rates remain largely unchanged. Triazole resistance is already widespread, and echinocandin resistance, while still low, is steadily climbing. The emergence of multidrug-resistant *Candida auris*, the growing immunocompromised population, and the absence of any antifungal vaccine collectively define this scenario as a therapeutic impasse. Given these challenges, there is an urgent need to synthesize current vaccine research to identify robust candidates and address lingering translational bottlenecks. This structured narrative review evaluates the advances and barriers in *Candida* spp. vaccine development over the past decade (2015–2025), structured around epidemiology and resistance; immunological framework underpinning vaccine design; antigenic targets and delivery platforms; and preclinical and clinical evidence. Key milestones included the Phase II clinical validation of NDV-3A, the first successful large-scale human trial of a fungal vaccine; the standardization of the A/J mouse model for *C. auris*; the emergence of multi-epitope computational platforms with >94% HLA population coverage; and cross-kingdom protection paradigms. Major obstacles included commensal-to-pathogen duality of *Candida*, immunocompromised host paradox, antigenic plasticity, and chronic underinvestment. Converging next-generation vaccine strategies are beginning to bridge the gap between bench discovery and clinical implementation.

## 1. Introduction

The global landscape of medical mycology is facing an unprecedented challenge with the rise in Invasive Candidiasis (IC), a severe systemic infection that continues to compromise patient outcomes worldwide. While *Candida albicans* historically dominated clinical isolation reports, the epidemiological paradigm has shifted drastically. Today, clinicians are increasingly confronting emerging Non-*Candida albicans Candida* Species (NCACs), most notably the highly resilient *Nakaseomyces glabrata* (formerly *C. glabrata*) and the multidrug-resistant *Candida auris*. In clinical settings, IC manifests primarily as candidemia (the invasion of the bloodstream) or as deep-seated infection within major organ systems. Rather than exogenous contraction, these life-threatening conditions typically stem from an endogenous source, where the host’s own commensal *Candida* populations exploit a compromised immune system to breach epithelial barriers [1]. In recent years, the incidence of invasive fungal infections has increased substantially, driven by the growing number of immunosuppressed individuals, the elderly, and patients in Intensive Care Units (ICUs) [1].

The global burden is stark. IC accounts for an estimated 250,000 to 700,000 cases annually worldwide, with mortality rates between 40% and 55% [2]. In developed countries, IC is the most common hospital-acquired fungal infection, contributing to more than 1.5 million annual deaths from invasive fungal infections [3]. Paradoxically, modern medical advancements have done little to curb the lethality of these infections. Despite progress in healthcare systems, intensive care, and microbiological techniques, mortality rates in IC remain largely unchanged [1]. Triazole resistance is increasing in several *Candida* species, particularly in high-risk clinical settings, while echinocandin resistance remains uncommon overall but has been reported with increasing frequency following prolonged or repeated exposure [4]. Our therapeutic arsenal is critically limited. Current management relies almost exclusively on conventional chemotherapy, which is frequently impacted by severe adverse effects and emerging resistance.

Beyond invasive disease, the global impact of *Candida* extends far beyond lethal systemic infections. Mucosal infections represent a massive, yet frequently underestimated, burden of morbidity. It is estimated that between 50% and 70% of healthy women worldwide will experience at least one episode of Vulvovaginal Candidiasis (VVC) during their lifetime [5]. More alarmingly, a substantial proportion (5% to 8%) of these patients develop Recurrent VVC (RVVC), characterized by four or more episodes per year, inflicting chronic suffering and severe socio-economic impact for which a definitive therapeutic solution is still lacking [5].

In this context, developing innovative preventive strategies, such as vaccines, is a clinical necessity [6]. However, no antifungal vaccine has yet been licensed for human use. Factors such as intra- and inter-species antigenic variations, a lack of commercial interest, and the immunocompromised profile of high-risk individuals hinder this process [7]. To address these gaps, this review evaluates the progress made and the barriers encountered in *Candida* vaccine development over the past decade, outlining the roadmap towards effective immunization.

This review is structured to cover the most clinically relevant axes: (i) epidemiology and escalating resistance; (ii) the immunological mechanisms underpinning protective immunity; (iii) key antigenic targets and delivery platforms; (iv) preclinical and clinical milestones; and (v) barriers and future directions. The objective is to provide a comprehensive, up-to-date synthesis for an audience at the forefront of infectious disease and vaccinology research.

## 2. Epidemiology, Resistance, and the Imperative for Vaccination

### 2.1. The Global Burden—A Rising Threat

Invasive fungal infections (IFIs) represent a critical threat to global public health, causing approximately 1.5 million deaths annually [8], an alarming toll that rivals the mortality of tuberculosis or HIV/AIDS [6]. Within this vulnerable population, *Candida* stands out as the primary pathogen, serving as the leading cause of nosocomial fungal bloodstream infections in Europe [8]. Recent global estimates suggest the true burden may be even heavier, reaching up to 3.8 million annual deaths, with 2.5 million directly attributable to fungal pathogens [3]. Yet, despite the mortality rate comparable to malaria, mycological research remains severely underfunded.

In the United States, the economic impact is estimated at $1.8 billion annually for approximately 22,000 infections [1]. On a global scale, 30-day mortality rates vary considerably, ranging from 38 to 42% in Europe to peaks reaching 70% in South America [1]. Recent studies [4] highlight significant regional variations in incidence: while in Europe rates range between 2.0 and 3.9 per 100,000 inhabitants, these figures are significantly higher in the United States, varying between 13.3 and 26.2 per 100,000 inhabitants. Table 1 summarizes the geographic variability in incidence and mortality.

It should be noted that the 40–55% global mortality range cited above and the regional South American estimate (up to 70%) derive from broader systematic literature reviews [1,2], whereas Table 1 reports 30-day mortality from specific national surveillance studies; the two are complementary rather than contradictory, reflecting different levels of geographic and methodological granularity.

### 2.2. The Shifting Landscape—From C. albicans to Non-C. albicans Candida Species

One of the most significant shifts over the last decade is the transformation in species epidemiology. While *C. albicans* largely dominated (70–80%) cases in the 1980s–1990s, its prevalence has currently dropped to 40–60% in favor of NCACs [1]. This transition is primarily driven by the selection pressure exerted by the extensive and prophylactic use of antifungals, especially fluconazole [4]. Crucially, this selective pressure has not only favored NCACs but has also fueled a concerning rise in azole resistance within *C. albicans* itself, challenging its status as an easily manageable pathogen. Beyond selective drug pressure, environmental catalysts (most notably climate change) are reshaping the mycological landscape. Global warming is hypothesized to have driven the thermal adaptation of previously environmental or non-pathogenic fungi, allowing them to breach the mammalian thermal barrier and emerge in geographic regions where they were historically absent. This dual pressure—epidemiological shift and climate-driven emergence—poses a critical biological barrier to the development of immunization strategies. Historically, most vaccine candidates have been designed based on *C. albicans*-specific antigens; however, due to significant genetic and phylogenetic divergence between species, antibodies and cellular responses induced against *C. albicans* often provide limited or no cross-protection against NCACs [1]. Table 2 details the emerging species and their resistance profiles.

### 2.3. Emerging Challenges—C. auris and Environmental Factors

The emergence of *C. auris* has become a critical concern for the World Health Organization (WHO). This pathogen stands out for its multidrug resistance to triazoles, echinocandins, and polyenes, with an associated mortality of 30% to 72% [9,10]. Resistance has reached critical thresholds: approximately 90% of isolates are resistant to fluconazole, 30% to amphotericin B, and 5% to echinocandins. Furthermore, up to 47% of isolates in some series also exhibit resistance to flucytosine (5FC) [3]. Its capacity to persist long-term on inanimate hospital surfaces radically complicates standard infection control measures.

Two exogenous factors compound this threat. First, the COVID-19 pandemic drove a severe surge in candidemia through prolonged ICU stays, increased use of invasive devices, and extensive corticosteroid and broad-spectrum antibiotic use—a phenomenon described as COVID-19-Associated Candidemia (CAC). Studies demonstrate the critical importance of response time: initiating antifungal treatment within the first 24 h raises the survival rate to 56%, compared to only 28% when treatment is delayed [7]. Second, climate change is hypothesized to force fungi to adapt to higher ambient temperatures, facilitating survival at human body temperature and favoring the emergence of thermotolerant species like *C. auris* [1]. This threat is further exacerbated by the massive use of azole fungicides in agriculture, creating a selection pressure that drives the emergence of resistant strains before any contact with the clinical environment [3].

### 2.4. Risk Factors and Host Vulnerability

ICU patients are the most vulnerable cohort, especially during prolonged stays exceeding 10 days. The Central Venous Catheter (CVC) is arguably the most critical risk factor, directly breaching the skin barrier (consequently, catheter removal is considered the gold standard for reducing mortality) [1]. Prolonged use of broad-spectrum antibiotics (exceeding 14 days) depletes the protective bacterial flora, while gastrointestinal surgeries or digestive perforations allow *Candida* species from the intestinal mycobiome to translocate into the peritoneal cavity or bloodstream [1,2]. Iatrogenic immunosuppression (corticosteroids, tacrolimus, cyclosporine) as well as haematological malignancies and solid tumor therapy with associated neutropenia and mucosal damage, constitute the core risk profile for invasive disease.

This clinical landscape creates the fundamental vaccinological paradox, often referred to in the literature as the “Catch-22” scenario: individuals at the most imminent risk of IC are, by definition, those who lack the cellular competence necessary to mount an effective adaptive immune response upon administration of a conventional vaccine [5]. Host genetic polymorphisms further modulate risk independently of treatment: mutations in STAT1 are associated with Chronic Mucocutaneous Candidiasis (CMC), while defects in CARD9 and SYK signaling pathways increase susceptibility to invasive infections [1]. Identifying patients with these specific genetic predispositions could pave the way for personalized vaccination strategies.

### 2.5. The Therapeutic Impasse—Why Vaccines Are the Next Frontier

The traditional approach based on antifungal agents such as polyenes, azoles, and echinocandins is reaching its limits. Breakthrough infections account for up to 40% of candidiasis cases in select high-risk cohorts. For instance, in lung transplant recipients, a documented 41% rate of breakthrough infections highlights the failure of current preventative pharmacotherapy [1]. Novel agents such as rezafungin, ibrexafungerp (the first oral triterpenoid), and fosmanogepix represent advances in therapeutics but their spectrum remains limited, global access is restricted, and cases of resistance are already surfacing [3].

Blood cultures, historically the diagnostic gold standard, detect only 17–50% of proven IC cases [1], creating a critical delay of 24–72 h before confirmatory results. Modern biomarkers such as (1,3)-β-D-glucan (BDG) or the T2 *Candida* test present a low positive predictive value in heterogeneous populations [2], fueling empirical over-treatment and further driving the cycle of host toxicity and drug resistance. An effective vaccine can induce robust local mucosal and systemic immunity to block fungal translocation at the gastrointestinal epithelium [2], neutralizing the fungus during its commensal phase through local Th17 cellular responses and secretory IgA (sIgA), and establishing an impassable frontline defense before deep tissue invasion can begin. This transition toward active immunization is justified by four pillars: stagnant mortality, the diagnostic bottleneck, the biomarker limitation and its economic burden.

## 3. Immunology of Candida Infections

### 3.1. The Commensal Paradox

Developing protective immunity against *C. albicans* represents a unique biological challenge due to the dual nature of this organism. As a symbiotic member of the human microbiota (gut, mouth, and skin), *C. albicans* normally coexist in homeostasis with the host, imposing a state of active immunological tolerance that prevents self-damaging inflammatory responses. However, any disruption, e.g., iatrogenic immunosuppression or physical breaches in epithelial barriers, forces the immune system to break this baseline tolerance and orchestrate a targeted attack against the now-pathogenic fungi. An effective anti-*Candida* vaccine should elicit protective immune responses directed against virulence-associated determinants, such as the yeast-to-hypha transition, while maintaining immune homeostasis and minimizing excessive inflammatory responses that could contribute to host tissue damage.

### 3.2. Innate Recognition—PRRs, Complement, and Signaling Cascades

The first line of defense against *Candida* species relies on the detection of conserved Pathogen-Associated Molecular Patterns (PAMPs) by Pattern Recognition Receptors (PRRs) on dendritic cells (DCs), macrophages, and neutrophils. C-type lectin receptors Dectin-1 and Dectin-2 serve as primary pillars. Dectin-1 specifically recognizes β-(1,3)-glucans, while Dectin-2 binds to surface mannans [11]. Different *Candida* species expose different cell wall ligands and therefore engage PRRs differently. While Dectin-1 plays a central role in the recognition of *C. albicans* and *C. tropicalis*, its relative contribution to the recognition of *C. glabrata* is less well established, reflecting species-specific differences in cell wall architecture and the absence of hyphal morphogenesis [12]. In *C. auris*, β-1,2-mannose linkages have been identified as the primary antigenic determinants, and the overall cell wall organization can actively hinder recognition by classical β-glucan-dependent PRRs [13]. Synergistic crosstalk between Dectin-1 and TLR2 creates an amplification cascade potentiating the production of pro-inflammatory cytokines such as TNF-α and IL-6.

The engagement of the Dectin-1/CARD9 axis activates NF-κB via Syk kinase and the CARD9/Bcl-10/MALT1 complex; TLRs utilize the MyD88/IRAK-1/4/TRAF-6 pathway to the same transcriptional endpoint [8]. A deficiency in CARD9 completely halts the production of key pro-inflammatory cytokines and blocks the differentiation of protective Th17 cells [11], rendering hosts extremely susceptible to *C. albicans*, *C. tropicalis*, and *C. glabrata* [12]. Recent functional studies [14] with *C. auris* have reaffirmed the indispensable role of innate immunity; a deficiency in the C5 complement component produces rapidly fatal systemic infection characterized by massive fungal proliferation in the brain, heart, and kidneys, without requiring chemical immunosuppression. The NLRP3 inflammasome, activated by filamentous fungal forms, drives the proteolytic release of active IL-1β and IL-18, orchestrating Th1 and Th17 differentiation; excessive activation may culminate in macrophage pyroptosis, paradoxically depleting vital sentinel populations and accelerating dissemination [15]. Table 3 summarizes these species-specific immunological divergences.

### 3.3. Adaptive Immunity—The Th1/Th17 Duality and Compartmentalized Protection

Protection against *Candida* is driven by the functional specialization of helper T lymphocytes (CD4+) (Figure 1). The Th1 response, triggered by IL-12 production by DCs, activating the phosphorylation of STAT1 and STAT4, and culminating in massive secretion of IFN-γ, remains the predominant mechanism controlling commensal populations within the gastrointestinal tract and combating systemic candidemia [8]. Recombinant IFN-γ as adjunctive therapy has successfully boosted macrophage clearance and reduced mortality in patients with refractory MDR fungal infections [9,11,15].

The Th17 arm is established as the primary orchestrator of mucosal and cutaneous immunity, driven by the synergistic actions of IL-6 and TGF-β activating STAT3 and RORγt [8,11]. Upon activation, Th17 lymphocytes secrete IL-17A, IL-17F, and IL-22; IL-17 acts as a potent chemoattractant in recruiting neutrophils, while IL-22 stimulates epithelial barrier reinforcement and antimicrobial peptide secretion, including defensins. Furthermore, IL-17 enhances the release of Neutrophil Extracellular Traps (NETs), immobilizing large extracellular hyphal filaments that escape phagocytosis [16]. The non-negotiable importance of this pathway is demonstrated by patients with Autoimmune Polyendocrine Syndrome Type 1 (APS-1), who carry neutralizing autoantibodies against IL-17 and systematically develop severe CMC [11]. Critically, the Th17 paradigm must be nuanced for NCACs: protective immunity against *C. tropicalis* in systemic infection relies far more on TNF-α than on IL-17 [12], demonstrating that exclusive Th17-focused vaccine strategies will fail to provide broad-spectrum coverage. Beyond initial polarization, vaccine design must account for cellular exhaustion during chronic infections.

Prolonged antigen exposure upregulates immune checkpoints. Targeting the PD-1/PD-L1 pathway has demonstrated potential to restore T-cell function and improve survival in vivo. This strongly suggests that next-generation vaccine strategies would benefit from adjuvant co-delivery designed to mitigate immune exhaustion.

The concept of compartmentalized protection poses a major hurdle to universal vaccine development. Mice immunized to mount an exclusive high-titer Th17 response achieve absolute protection against cutaneous challenges but remain highly vulnerable to fatal candidemia. Conversely, a vaccine driving a robust Th1 response guarantees host survival against systemic candidemia but proves incapable of clearing skin niches [17]. Vaginal mucosa is a notable exception: unlike the oral cavity, vaginal protection appears less dependent on adaptive T lymphocytes and relies more on innate mechanisms and alarmins [11], explaining why candidates effective against candidemia frequently fail to prevent VVC.

### 3.4. Morphological Plasticity and Immune Evasion Strategies

The pathogenicity of *Candida* spp. is driven by active, dynamic manipulation of the host’s cellular components, regulatory pathways, and immune recognition mechanisms. During the yeast-to-hypha transition, highly immunogenic β-glucans are strategically buried beneath a dense outer cloak of mannoproteins [17], preventing Dectin-1 recognition and silencing the immunological alert required to initiate protective Th17 differentiation.

Secreted Aspartyl Proteases (SAPs) simultaneously degrade complement proteins and immunoglobulins (IgA and IgG), neutralizing the antibody-mediated response before it reaches the fungal surface (Figure 2) [16]. Filamentous forms can actively skew host defenses toward a Th2 response (via IL-4) correlated with fungal persistence and drive the expansion of Treg cells that suppress adjacent Th1 and Th17 effector responses, shifting the host immune balance toward tolerance [11].

An innovative dimension of *Candida* pathogenesis involves extracellular Hsp90 (eHsp90). Unlike conventional virulence factors, eHsp90 is released as a collateral product of fungal lysis, typically induced by an initial macrophage attack or antifungal treatment [18]. Once released, eHsp90 binds TLR4, primes NF-κB, and accelerates the aberrant activation of the NLRP3 inflammasome and caspase-1, ultimately driving widespread macrophage pyroptosis that decimates vital immune sentinel populations and worsens functional pathogen loads in target organs [18]. Studies of a specific 47kDa fragment showed that antibodies targeting this fragment can neutralize the hyperinflammation without affecting host functions, validating eHsp90 neutralization as a synergistic counterstrategy to complement active vaccination [17,18].

Species-specific evasion strategies also diverge markedly. *C. glabrata*, lacking the genetic machinery to undergo hyphal filamentation, utilizes a “Trojan horse” strategy; it survives and replicates silently within macrophages without triggering a vigorous inflammatory response [12]. This intracellular persistence demands a much more potent macrophage activation via Th1/IFN-γ than required for other species, a distinction with direct implications for species-specific vaccine design.

### 3.5. Humoral Immunity—A Complementary but Essential Role

While cellular immunity remains the cornerstone of antifungal defense, the humoral response exerts a critical, multifaceted influence. Beyond classical opsonization via Fc and CR3 receptors, distinct antibodies exhibit direct candidacidal activity, independently inhibiting fungal growth and biofilm formation, and blocking Als3p-mediated adhesion and the morphological transition to the hyphal form. Simultaneously, the humoral response neutralizes SAPs, preventing degradation of tissue barriers and defense immunoglobulins [19].

B lymphocytes also act as fundamental APCs, shaping the magnitude of downstream adaptive responses by secreting cytokine profiles that guide Th1 and Th17 differentiation [16]. Developing conjugate vaccines is therefore essential to shift the natural transient IgM response toward high-affinity IgG production, securing immunological memory. A recent work identified disaccharide 19 (β-1,2-mannose disaccharide) as the minimal immunodominant epitope in *C. auris* [13], enabling development of diagnostic tools capable of detecting the pathogen as early as the third day after infection.

### 3.6. Summary—Immunological Levers and Biological Barriers

The tension between host protective mechanisms and the survival strategies of *Candida* underscores a fundamental truth: vaccine success depends not only on antigen immunogenicity but also on its ability to precisely guide the immune response according to the targeted anatomical compartment. Next-generation vaccines must adapt to species heterogeneity. Effector mechanisms and molecular targets differ radically across the *Candida* genus [12]. Vaccine efficacy will depend on the induction of cross-reactive antibodies recognizing conserved epitopes in NCACs such as *C. auris* or *C. glabrata*, compensating for cellular immunity failures in these species. Table 4 summarizes these host–pathogen dynamics.

## 4. Vaccine Targets, Antigens, and Delivery Platforms

### 4.1. Specific Targets and Surface Proteins

Research focuses heavily on proteins essential for fungal virulence, specifically cell-wall adhesins from the Als family and SAPs [6,7]. The one leading candidate is the NDV-3A vaccine, built from the recombinant N-terminus of Als3p (rAls3p-N). Beyond single proteins, efficacy can be amplified through molecular fusions: the AH-AIPO platform physically fuses key segments of Als3 and Hyr1p into a single recombinant construct, blocking both initial biofilm assembly and subsequent systemic dissemination [10]. For *C. tropicalis*, the Sap2 protease is a recognized vaccine target; immunoinformatic screens identified 11 conserved, non-allergenic epitopes with less than 30% sequence homology to human proteases [21]. The conserved glycolytic enzyme Fba1, stably expressed in both yeast and hyphal stages, offers an epitope (YGKDVKDLFDYAQE) that cannot be masked during morphological transition, limiting fungal escape capacity [22].

For *C. auris*, a fundamental paradigm shift was required. Subtractive proteomics, filtering over 5500 *C. auris* proteins, identified novel antigens lacking human homologs: Uricase, SUN family proteins, and specific mitochondrial import receptor subunits [23]. A separate reverse vaccinology pipeline identified 25 candidates with high antigenicity, structural stability, zero human/microbiome homology, and estimated 94% HLA class I allele population coverage [24].

### 4.2. Polysaccharide and Glycan-Based Targets

The *Candida* cell wall is rich in structural carbohydrates, primarily β-glucan and mannans, whose high surface abundance makes them ideal antibody targets. However, isolated fungal polysaccharides are naturally poorly immunogenic because they fail to activate T-lymphocyte pathways alone [6,7]. Conjugate vaccines chemically link target polysaccharides to immunogenic carrier proteins (KLH, CRM197), successfully converting the response from T-cell independent to T-cell dependent, generating high-affinity IgG antibodies and durable immunological memory [6,7]. The latest frontier involves precision synthetic glycans: for *C. auris*, structural mapping confirmed β-1,2-mannans as the dominant antibody targets, with disaccharide 19 identified as the minimal immunodominant epitope [13]. Engineering of synthetic β-glucan oligosaccharides branched with 6-O-linkages demonstrates that immunogenicity depends on the size and density of lateral branches rather than linear chain length, enabling self-adjuvating trisaccharide conjugates that induce strong humoral immunity without external adjuvants [25].

### 4.3. Vaccine Platforms and Delivery Systems

Whole-cell vaccines are also part of the *Candida* vaccine development pipeline. Live attenuated strains (tet-NRG1, PCA-2, CNC13) closely mimic natural infection, triggering broad cellular and humoral activation with rapid PMN recruitment [6,7,10]. Inactivated formulations offer superior safety but require external adjuvants; combining inactivated *C. albicans* and *C. tropicalis* cells drives an eightfold increase in specific antibody titers following a secondary booster dose [26]. *Candida* Ghosts (CGs), hollow cellular envelopes emptied via the Sponge-like Reduced Protocol (SLRP) while preserving native cell wall architecture, trigger a high-fidelity immune response that closely mirrors natural host defense mechanisms without any risk of fungal replication [27]. Proteomic analysis of Cell Wall Surface Protein (CWSP) extracts reveals a complex mix of ~20 antigenic proteins, among which Mp58 (Prap1p, pH-regulated antigen 1) stands out as highly abundant and immunodominant, stimulating multiple immune pathways simultaneously [28].

To enhance the stability, targeted release, and overall efficacy of these traditional formulations, the field is rapidly shifting toward advanced delivery systems. Thermosensitive chitosan hydrogels (CH-HG) function as smart sustained-release matrices; via a “proton sponge effect”, buffering the acidic endosomal environment, inducing membrane rupture, and allowing antigen cytosolic escape, they significantly reduce the need for multiple booster doses [20]. DODAB:MO liposomes mimic natural fungal particulate geometry, prolonging DC activation and driving persistent IgG1/IgG2a responses; the ADS1 formulation achieved 62.5% survival in murine IC models [29]. The Spontaneous Nanoliposome Antigen Particle (SNAP) platform incorporates cobalt-porphyrin-phospholipid conjugates alongside MPLA and QS-21, allowing polyhistidine-tagged peptides to spontaneously anchor to vesicle surfaces and triggering specific IgG titers significantly higher than traditional conjugate designs at a ten-fold lower peptide dose [30]. Plasma Beads (PB-PLGA) use biodegradable microspheres embedded within a blood plasma matrix, providing strict kinetic control over antigen release that mimics continuous pathogen exposure without live infection risk [31].

Alongside these traditional cellular approaches, the field has entered the era of next-generation vaccinology through the exploration of nucleic acid platforms. DNA and mRNA vaccines can co-express immunomodulatory molecules such as IFN-γ as a built-in self-adjuvating system, actively directing a weakened host immune system toward protective Th1/Th17 differentiation and bypassing the chronic immune silencing found in immunocompromised patients [32]. Phage display presents specific epitopes on bacteriophage coat proteins (pIII or pVIII), functioning as immunogenic nanoparticles with high thermal stability and low production cost, a scalable platform for developing nations where cold-chain logistics are a critical obstacle [22]. Fungal extracellular vesicles (EVs) naturally package complex immunogenic wall proteins and virulence factors, possessing intrinsic capacity to mature DCs in vitro and orchestrate balanced humoral and cytokine responses in vivo, with preserved membrane integrity even after prolonged storage at −20 °C and −80 °C [33]. Bacterial vectors like genetically modified *Lactobacillus* carry *Candida* genes directly to the intestinal mucosa, establishing protective immune barriers precisely at the primary gastrointestinal entry points of the commensal fungus [7]. Figure 3 summarizes this information.

### 4.4. Multivalent Vaccines and Cross-Protection

A major paradigm shift is the transition from species-specific formulations to broad-spectrum, multi-target solutions. Combining purified protein extracts from *C. albicans* (3 mg/mL) and *C. tropicalis* (5 mg/mL) drives humoral antibody responses equivalent to crude whole-cell vaccines, reaching specific titers of 1:3200, with superior product purity and a significantly lower risk of adverse inflammatory reactions [26]. Computer-aided design (CAD) enables engineering of consensus vaccines by identifying conserved epitopes shared across phylogenetically distant species, ensuring simultaneous coverage of susceptible *C. albicans* and highly resistant *C. auris* [23]. Furthermore, the NDV-3A platform has demonstrated cross-kingdom protection. Als3p shares structural similarities with specific bacterial surface adhesins, meaning vaccine-induced antibodies also provide cross-protection against *Staphylococcus aureus* infections [6,7,10].

## 5. Preclinical Advances and Clinical Trials

### 5.1. Computational Validation

The last decade has marked a fundamental shift toward precision molecular design supported by bioinformatics. Protein-protein docking and molecular dynamics simulations, typically conducted over 100-nanosecond trajectories, verify high-affinity interactions between engineered vaccine constructs and host PRRs (TLR4, TLR5), and confirm structural stability under physiological conditions at 37 °C via Root Mean Square Deviation (RMSD) analysis [23,24,34]. In silico immune simulations predict downstream cytokine production and forecast the generation of long-lasting immunological memory [23,24]. This approach has recently been consolidated by the development of an innovative dual-target MEVC against HPV-16 and *C. albicans*. Molecular docking and explicit dynamics simulations demonstrated strong binding affinities within human MHC class I and II grooves, strongly suggesting low risks of allergenicity and toxicity before laboratory synthesis [35].

### 5.2. Key Preclinical In Vivo Evidence

Active immunization with NDV-3A confers robust cross-protection against *C. auris* in chemically immunosuppressed mice, reducing renal and brain fungal loads by approximately 10-fold, increasing survival to 40% where control mortality reached 100%, and demonstrating therapeutic synergy when co-administered with micafungin [36]. Vaccine-generated anti-rAls3p-N antibodies inhibit *C. albicans* adherence and endothelial cell invasion by 50%, reduce initial biofilm formation on catheter surfaces by an equal margin, and reduce the mechanical dissemination of yeast cells from mature hyphal layers by 35%, effectively blocking the primary pathway driving nosocomial hematogenous dissemination [37].

The bivalent peptide candidate Fba-Met6 (MP12) maintains high antibody titers up to 5 months post-immunization; pharmacological depletion of complement proteins using Cobra Venom Factor completely abolishes its protective efficacy, establishing a vital mechanistic link between vaccine-induced IgG1/IgG2a and innate complement activation [38]. Native *C. albicans* EVs conferred absolute protection (100% survival) in severely neutropenic murine models, with statistically significant CFU reductions across the liver, spleen, and kidneys. Thus, establishing EVs as premium candidates for neutropenic patient populations [33]. Figure 4 summarizes the preclinical survival spectrum.

### 5.3. The A/J Mouse Model Breakthrough and Route of Administration

One of the major preclinical advances is the standardization of the A/J mouse strain as the preferred model for *C. auris* [14]. This species possesses a natural genetic deficiency in the C5 complement component, rendering it highly susceptible to fatal systemic infection with 100% mortality without immunization and without requiring chemical immunosuppression that masks true vaccine-induced responses. This allows survival and fungal clearance results to be directly attributed to immunological memory. The FM-SNAP bivalent platform demonstrated remarkable prophylactic efficacy in this model, producing statistically significant reductions in renal fungal load against MDR *C. auris* challenges [30].

Recent preclinical evidence further demonstrates that the route of administration is a critical determinant of vaccine outcomes [28]. Mucosal immunization routes (intravaginal, intranasal), while intuitively suited to localized pathologies, frequently fail to induce durable systemic immunological memory compared to parenteral approaches. Critically, vaccine formulations that achieve protective efficacy exceeding 60% via intradermal or intramuscular injection result in absolute therapeutic failures when applied to mucosal sites [28]. Standardizing delivery routes with focus on antigen-loaded DC biodistribution to regional lymph nodes is therefore a foundational directive for next-generation *Candida* vaccines.

### 5.4. Trained Immunity and Myelopoiesis Reprogramming

A foundational paradigm shift is the recognition that vaccine protection relies not solely on adaptive T and B cells but also on long-term innate immune reprogramming, i.e., trained immunity. Using single-cell RNA sequencing (scRNA-Seq), exposure to the live-attenuated strain PCA-2 was shown to trigger emergency myelopoiesis, functionally reprogramming hematopoietic stem and progenitor cells (HSPCs) in the bone marrow via stable epigenetic histone modifications and increased glycolysis [6,10,29,40]. These reprogrammed HSPCs migrate temporarily to the spleen and differentiate into monocytes and macrophages with enhanced capacity to clear fungal cells and secrete protective TNF-α and IL-6 [40]. Crucially, autocrine GM-CSF was identified as the mandatory molecular mediator. Blocking it in vivo completely prevents HSPC mobilization and abolishes the protective effect of the vaccine [40], opening new paths for designing targeted adjuvants that interact directly with bone marrow progenitor cells.

### 5.5. Clinical Trials: NDV-3A and PEV7

The most advanced candidate is the NDV-3A vaccine, based on rAls3p-N, which successfully completed Phase Ib and Phase II clinical trials, proving safe and highly immunogenic in humans [34,41]. NDV-3A induces a dual protective mechanism: a humoral response generating antibodies that block fungal adherence, combined with a robust cellular response characterized by IFN-γ and IL-17A production by T lymphocytes [15]. In a randomized, double-blind, placebo-controlled Phase II trial involving 188 patients, while the global cohort analysis (ages 18–55) showed a positive trend without reaching full statistical significance, the subgroup of women under 40 years demonstrated high vaccine efficacy. Vaccination doubled the median time to the first symptomatic episode (210 days versus 105 days in placebo) and increased the recurrence-free rate at 12 months to 42%, compared to only 22% in placebo [41]. The vaccine maintained a highly favorable safety profile, with adverse events restricted to mild, transient local reactions at the injection site. This trial marks the first successful large-scale clinical validation of a fungal vaccine [6,34,41]. Table 5 summarizes the key clinical outcomes.

Beyond *Candida*, NDV-3A offers cross-kingdom protection. Vaccine-induced antibodies cross-protect against *S. aureus* infections [6]. Recent studies have expanded this to cross-protection against systemic *C. auris* challenges [36]. In parallel, the PEV7 candidate used virosomes to deliver recombinant Sap2 via intravaginal administration. Phase I trials confirmed complete safety, tolerability, and mucosal immune response generation without systemic side effects [6,34]. However, demonstrating any vaccine efficacy in profoundly neutropenic populations remains a major challenge, as the clearance of antibody-opsonized *Candida* cells relies heavily on functional innate effector cells, particularly neutrophils [15].

## 6. Major Barriers to Development

### 6.1. The Commensalism Dilemma and Fungal Plasticity

A successful vaccine must distinguish between benign colonization and active pathogenic infection. The generated immune response must target and eliminate invasive forms without destroying *Candida* role as a natural component of the human microbiota [6,7]. An excessively broad immune response risks total fungal eradication, triggering severe gut dysbiosis and metabolic imbalance that would turn a prophylactic vaccine into a secondary cause of severe microbiome disruption [6,7,42]. Vaccine designs must therefore focus specifically on morphological virulence traits such as filamentation rather than clearing the species entirely.

*Candida* spp. possesses remarkable genetic and morphological plasticity, continuously transitioning between yeast and hyphal forms, which means surface antigens displayed change radically depending on morphological state [6,7]. During the transition to hyphae, *Candida* activates virulence machinery including SAPs and candidalysin, a cytolytic peptide toxin that actively destroys host epithelial barriers, while hiding β-glucan beneath mannoproteins, creating a formidable barrier against vaccine-induced antibodies [42]. Under selection pressure from host antibodies, *Candida* can mask critical epitopes [43]. Targeting highly conserved essential enzymes such as Fba1, stably expressed in both yeast and hyphal stages, limits the fungus’s evasion capacity, but achieving complete protection still requires combining multiple targets to overcome functional redundancy [22].

### 6.2. The Immunocompromised Host Paradox

The transition of *Candida* vaccines to clinical practice faces a fundamental conceptual barrier known as “the host paradox”: patient populations that urgently need vaccine protection such as, chemotherapy patients, transplant recipients, HIV-positive individuals, ICU patients, are precisely those with the most fragile immune systems [1,15,44]. Live-attenuated strains carry a prohibitive risk of reversion to pathogenicity in immunodeficient hosts. In ICU patients with complex pathophysiology of multi-organ failure, inducing a stable immune response is an almost insurmountable clinical challenge [45]. A severe shortage of final effector cells, such as in profound neutropenia, can cause conventional vaccination to fail completely [15].

Beyond treatment-induced immunosuppression, intrinsic host immunogenetic variability acts as a silent biological barrier. SNPs in *CLEC7A* (Dectin-1) and cytokine signaling genes (IL-6, IL-17, STAT3) determine susceptibility independently of treatment [46], meaning congenital defects in immune machinery can prevent effective defense even with a promising candidate. Nucleic acid-based platforms (DNA and mRNA) offer an innovative bypass: co-expressing immunomodulatory cytokines inside host cells, they actively reprogrammed the weakened immune microenvironment, forcing Th1/Th17 differentiation and bypassing chronic immune silencing [32]. The use of thermosensitive chitosan hydrogels represents a complementary strategy, promoting endosomal escape via the proton sponge effect, allowing moderate antigen doses to successfully trigger essential Th1 and Th17 pathways even when conventional immune signaling is suppressed [20].

### 6.3. Immunological Safety, Autoimmunity, and Model Limitations

When a highly potent vaccine overstimulates a weakened immune system, the sudden influx of cytokines (IFN-γ and TNF-α) can cause severe collateral tissue damage known as Immune Reconstitution Inflammatory Syndrome (IRIS) [15,42]. Any candidate inducing strong cellular stimulation without strict regulatory feedback mechanisms risks mimicking the hyperinflammatory profile driven by NLRP3 hyperactivation that is responsible for much IC-associated tissue pathology [42]. The risk of vaccine-induced autoimmunity through molecular mimicry is inherent when targeting conserved proteins such as Hsp90, essential in both fungal and human eukaryotic cells, making exhaustive bioinformatic epitope mapping mandatory before human trials [43]. Non-mammalian models (*Caenorhabditis elegans*, *Galleria mellonella*) possess only innate immune defenses and entirely lacking adaptive immunity. Consequently, they cannot be used to evaluate active immunization or long-term immunological memory, creating an exclusive and expensive dependence on mammalian models [47].

### 6.4. Clinical, Regulatory, and Market Barriers

Blood culture-based diagnosis has limited sensitivity (approximately 17–50%) and often requires 24–72 h for pathogen detection, contributing to delays in definitive diagnosis and complicating the definition of precise clinical endpoints in vaccine efficacy trials [1,45]. In addition, the heterogeneity of target populations, comprising distinct groups of immunocompromised patients such as those with neutropenia, malignancy, or transplant-associated immunosuppression, poses significant challenges for the standardization of clinical trial design and may complicate regulatory evaluation across FDA and EMA frameworks [43]. Furthermore, the incorporation of advanced delivery platforms, including nanoparticles and hydrogel-based systems, introduces additional regulatory considerations. Beyond manufacturing complexities, these novel platforms carry specific safety limitations, such as potential local reactivity, systemic toxicity, or unpredictable long-term side effects, which demand extended preclinical and clinical safety data and significantly prolong translational and approval timelines [10]. From a macroeconomic perspective, pharmaceutical industry underinvestment in fungal vaccines relative to viral and bacterial platforms is chronic and structural [34]. The massive financial costs of expressing, purifying, and quality-controlling complex eukaryotic macromolecules are paired with the perception of an uncertain market return from fragmented, high-risk patient niches rather than the healthy global public. This financial bias perpetuates chronic underfunding, systematically blocking top-tier academic candidates from entering final-phase human testing [34].

## 7. Novel Approaches and Future Directions

### 7.1. Next-Generation Vaccinology and Glyco-Engineering

The future of immunization against *Candida* relies on reverse vaccinology approaches and structural immunoinformatics [23,34]. Subtractive proteomics based on genome-scale datasets enables in silico identification of potential antigenic targets, reducing reliance on pathogen culture, although experimental validation remains essential [23]. Chimeric multi-epitope vaccine designs can be optimized to achieve high predicted HLA population coverage across diverse human populations and are often produced using recombinant expression systems such as *E. coli* [21,24]. Emerging glyco-engineering strategies may modulate antigen immunogenicity, potentially influencing Th1/Th17-associated cytokine responses such as IFN-γ and IL-17, although these effects are context-dependent and not universally established [28].

### 7.2. Pan-Fungal, Multivalent, and Cross-Kingdom Strategies

Research is shifting toward multivalent “pan-fungal” vaccines capable of protecting against a wide range of *Candida* species as well as other opportunistic fungi such as *Aspergillus* and *Cryptococcus* [6,34]. Developing multivalent pan-fungal strategies is the most viable response to bypass the multifactorial mechanisms of antifungal resistance, closing evolutionary escape pathways by forcing the immune network to recognize multiple essential fungal targets simultaneously [48]. The cross-kingdom paradigm extends further: Hyr1p shares striking three-dimensional structural homology with virulence antigens on MDR Gram-negative bacteria *A. baumannii* and *K. pneumoniae* (FhaB and OmpA proteins) [49]. Monoclonal IgM antibodies directed against a specific immunodominant Hyr1p epitope conferred robust protection, survival rates of 70–100%, in immunocompetent and neutropenic mice against lethal pulmonary challenges with these bacteria, with drastic reductions in lung bacterial burdens [49].

### 7.3. Trained Immunity, Precision Adjuvants, and Passive Strategies

The BCG vaccine-based trained immunity strategy of inducing epigenetic and metabolic reprogramming in innate monocytes and NK cells creates a durable, non-specific line of defense that narrows the therapeutic window in critically ill patients before adaptive memory develops [7,34]. Precision adjuvant design maps individual immunogenetic profiles to compensate for specific molecular immune defects: patients carrying SNPs that selectively impair Th17 signaling can receive vaccines formulated with adjuvants engineered to over-activate that specific deficient pathway [46]. Plant-derived phytocompounds such as, ginsenosides, *Astragalus* and *Ganoderma lucidum* polysaccharides, berberine, curcumin, rosmarinic acid, and geraniol offer effective immune reinforcement with lower toxicity than aluminium salts, while simultaneously inhibiting biofilm formation and the yeast-to-hypha transition [50].

For profoundly immunocompromised patients, passive immunization offers the most immediate clinical pathway. Efungumab (Mycograb), a human-derived scFv targeting fungal Hsp90, when combined with Amphotericin B, disrupts the fungus’s molecular stress response and has significantly elevated survival rates in clinical trials (Table 6). Other innovative fronts include CAR-T cell therapies engineered for fungal targets, adoptive transfer of antigen-specific CD4+ T cells targeting the cell wall protein Ccrf1, PD-1 blockade to reverse T-cell exhaustion during severe infections, and recombinant cytokines (G-CSF/Filgrastim, IFN-γ) alongside donor granulocyte transfusions for deep neutropenia [9,15].

### 7.4. New Antifungal Agents and Global Health Integration

A new generation of antifungals addresses PK/PD limitations of conventional classes. For example, rezafungin’s prolonged half-life enables once-weekly dosing (STRIVE study); ibrexafungerp provides excellent oral bioavailability and efficacy against azole-resistant *C. glabrata* (MSG-10 study). Fosmanogepix achieves exceptional penetration into CNS and vitreous sanctuary sites via Gwt1 enzyme inhibition. Nikkomycin Z provides species-specific fungicidal activity by targeting chitin synthase, absent in human cells [3,9,10]. The clinical introduction of these new classes represents a historic milestone, offering optimized PK/PD profiles that overcome the toxicity limitations historically associated with conventional antifungals in critically ill patients [45].

Future directions converge toward global health integration. The One Health approach of coordinating agricultural fungicide use with medical antifungal stewardship is now considered imperative to preserve the clinical efficacy of newly developed drug classes [3,13]. Closing the gap in antifungal vaccine development requires biomedical science to prioritize thermostable formulations and economically sustainable bio-manufacturing processes, ensuring that biotechnological innovation translates into genuine therapeutic equity on a global scale [28].

## 8. Methods

The selection of articles included in this review was conducted in accordance with the Preferred Reporting Items for Systematic Reviews and Meta-Analyses (PRISMA-informed narrative synthesis) guidelines (Figure 5). The search was performed across the PubMed and ScienceDirect databases, using a search strategy combined with Boolean operators. Search terms combined “Candida” or “Candida auris” with terms specific to each of the three thematic pillars addressed here: for structural and clinical barriers, “translational barriers,” “antifungal resistance,” “regulatory,” and “clinical trial design”; for emerging immunotherapeutic strategies, “passive immunization,” “monoclonal antibody,” “dendritic cell,” “cytokine adjuvant,” “nanovaccine,” and “antifungal agent”; and for the One Health dimension, “One Health,” “diagnostics,” “HLA,” and “global health equity,” each combined with “fungal” or “antifungal”.

The inclusion criteria were the following: original articles or reviews published in peer-reviewed journals; studies addressing the development, pre-clinical trials, or clinical trials of *Candida* vaccines; articles published in English; publication date between 1 January 2015, and 31 December 2025 (Table 7). The exclusion criteria were: opinion pieces, editorials, letters to the editor, and conference abstracts; studies that did not present original data on vaccines; duplicate publications.

Of 222 articles identified (ScienceDirect n = 157; PubMed n = 65), 17 duplicates were removed, leaving 205 articles screened against title and abstract; 113 were excluded as irrelevant at this stage, leaving 92 records assessed for full-text eligibility. After full-text review: 1 not published in English was excluded; 1 with outdated data was excluded, and 36 were excluded due to a lack of novel insight relevant to the research question or redundant findings with already included literature. Finally, 54 articles were included in the final review.

## 9. Conclusions

Current research highlights a significant shift in the development of therapeutic and prophylactic strategies against IC. Confronted by a rising global tide of MDR species and strains, the field has successfully transitioned from classical empiricism to data-driven discovery. The integration of reverse vaccinology and immunoinformatics has bypassed the limitations of whole-cell extracts, allowing the design of highly precise recombinant expression subunits and multi-epitope vaccines with extensive global population coverage.

A major advance shown in this work is the precise orchestration of host defense mechanisms. Rather than driving generic inflammation, next-generation vaccine candidates specifically trigger the Th1 and Th17 immunity axes, which are now recognized as essential for clearing systemic *Candida* infections. When combined with advanced delivery systems, like biomimetic nanoparticles and trained immunity protocols, these strategies offer a crucial line of defense capable of overcoming immune paralysis even in vulnerable, highly susceptible patient populations. These biotechnological milestones, ranging from the Phase II clinical validation of the NDV-3A vaccine to multi-antigen pan-fungal platforms, demonstrate that a protective vaccine is approaching clinical feasibility: the NDV-3A Phase II trial provided the first proof-of-concept efficacy signal in a pre-specified subgroup (women under 40 years), although the global-cohort endpoint did not reach full statistical significance. Moving forward, the global impact of these advances will depend on addressing translational bottlenecks. Closing the gap between academic discovery and clinical implementation requires transitioning toward thermostable, cost-effective, and globally accessible formulations, ensuring that cutting-edge innovation translates into genuine therapeutic equity worldwide.

## Figures and Tables

**Figure 1:**
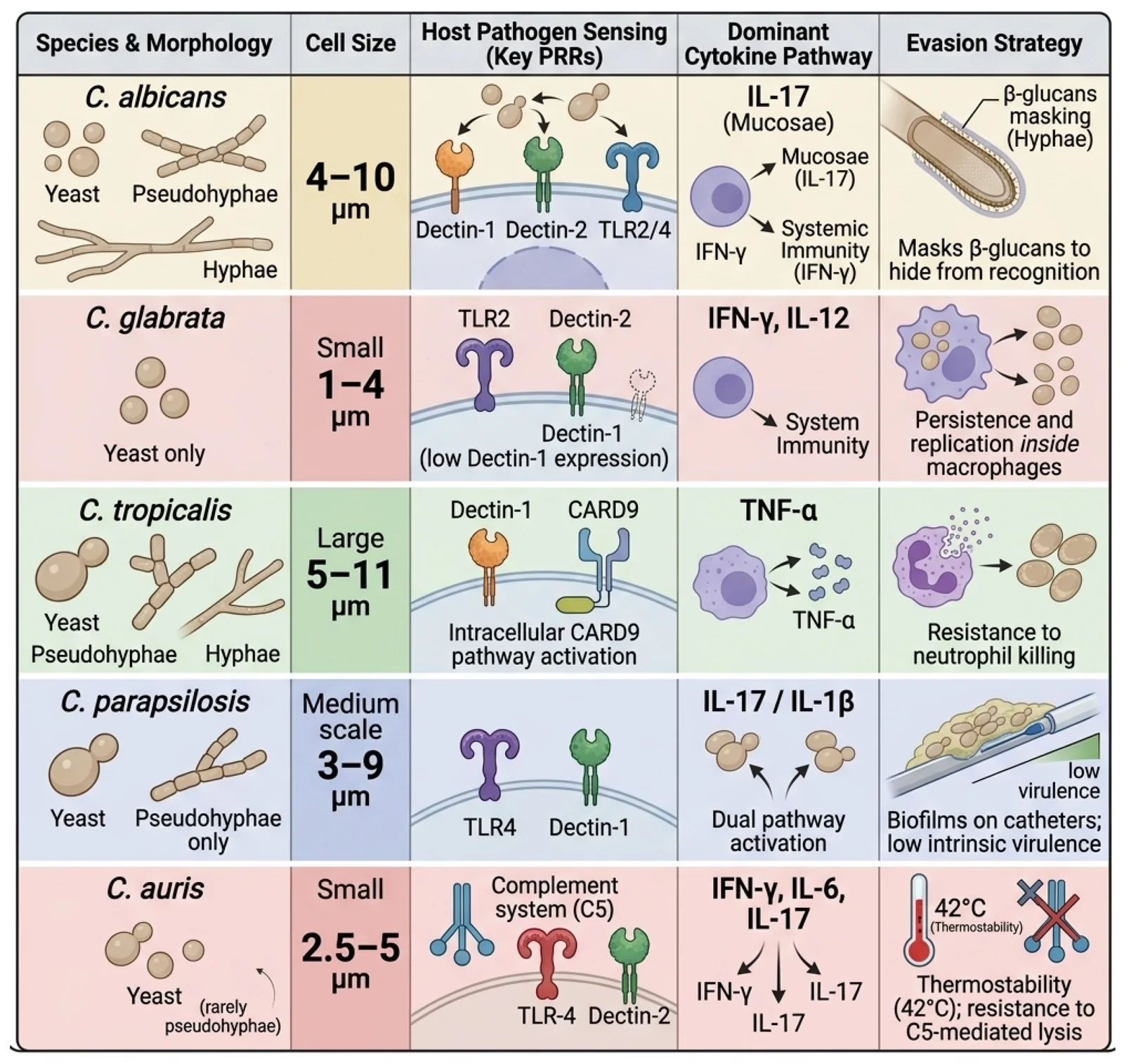
Phenotypic and Immunological Divergences among major *Candida* species [8,11,12,13,14].

**Figure 2:**
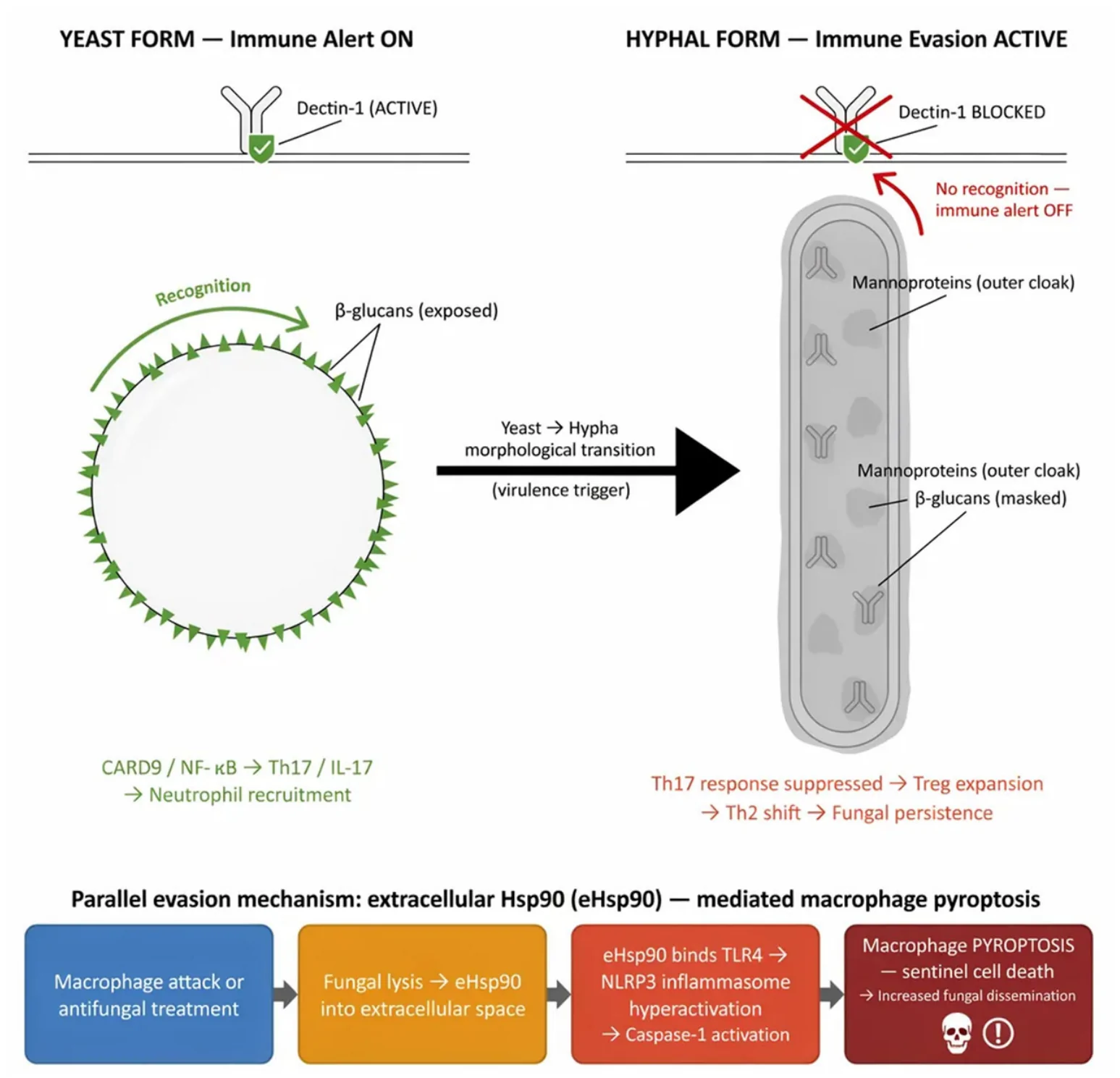
Morphological Plasticity and Immune Evasion—*Candida* Dual Attack [17,18].

**Figure 3:**
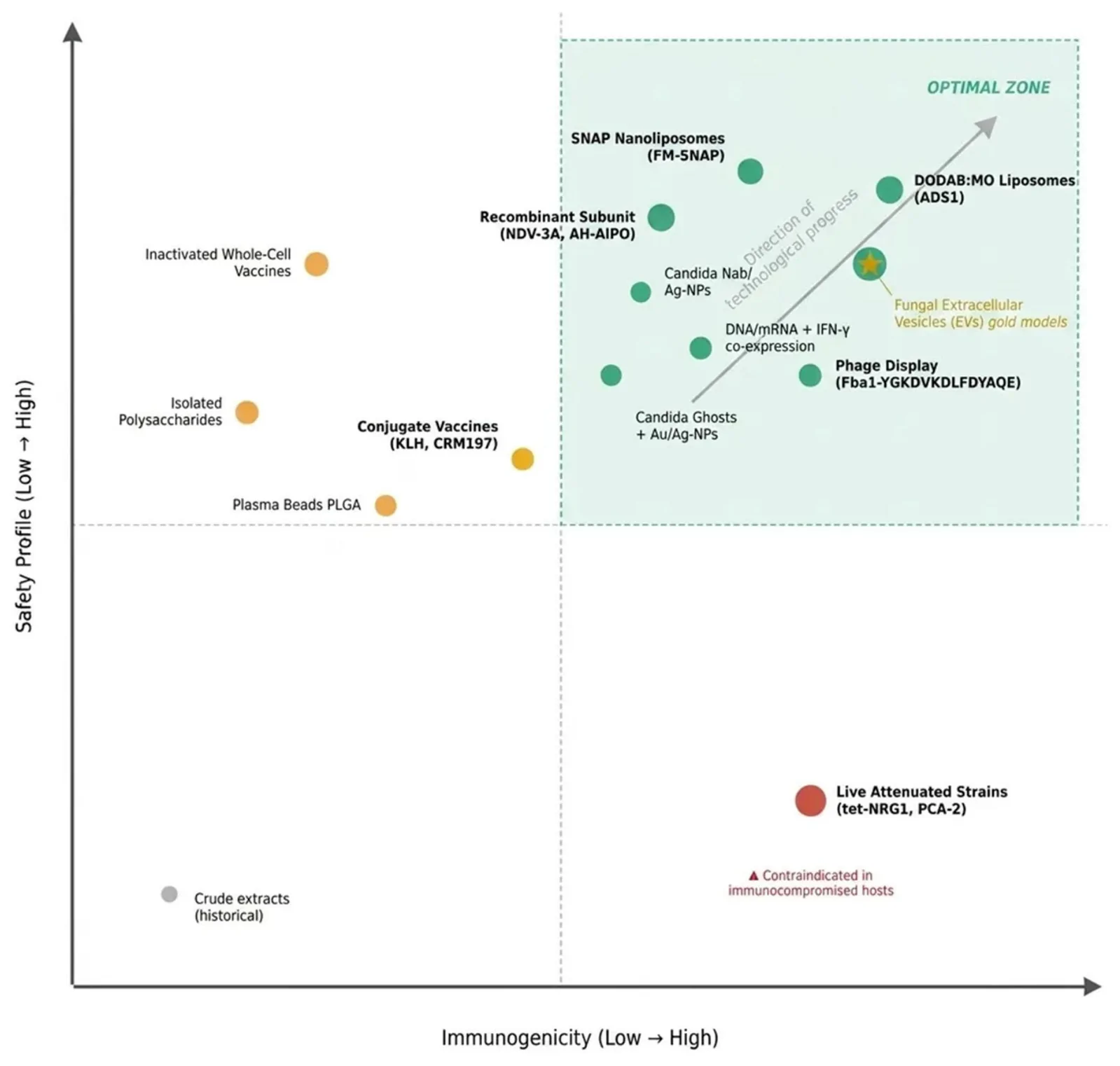
Vaccine Platform Selection Matrix: Safety vs. Immunogenicity.

**Figure 4:**
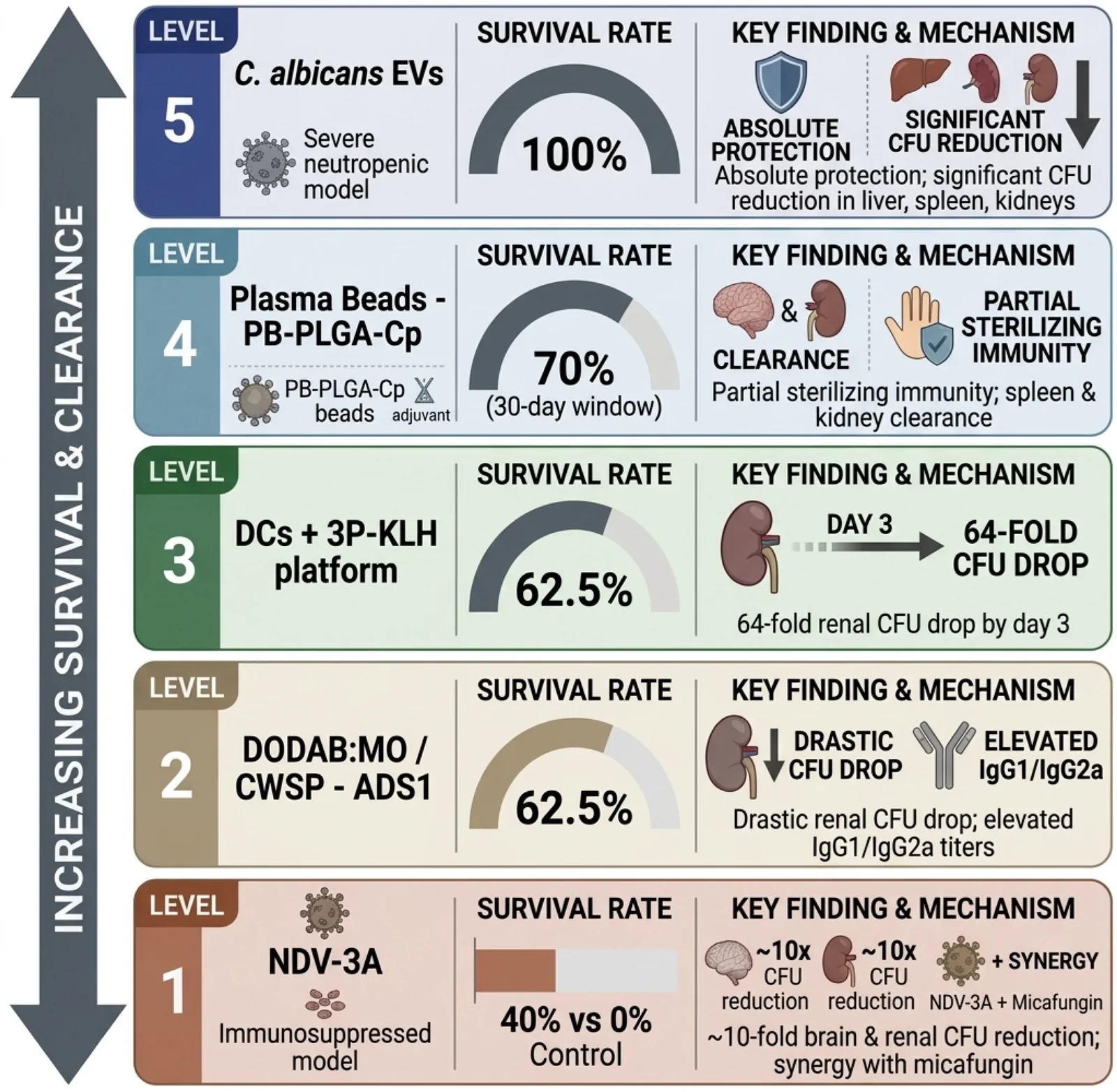
Preclinical survival efficacy spectrum [29,31,33,36,39].

**Figure 5:**
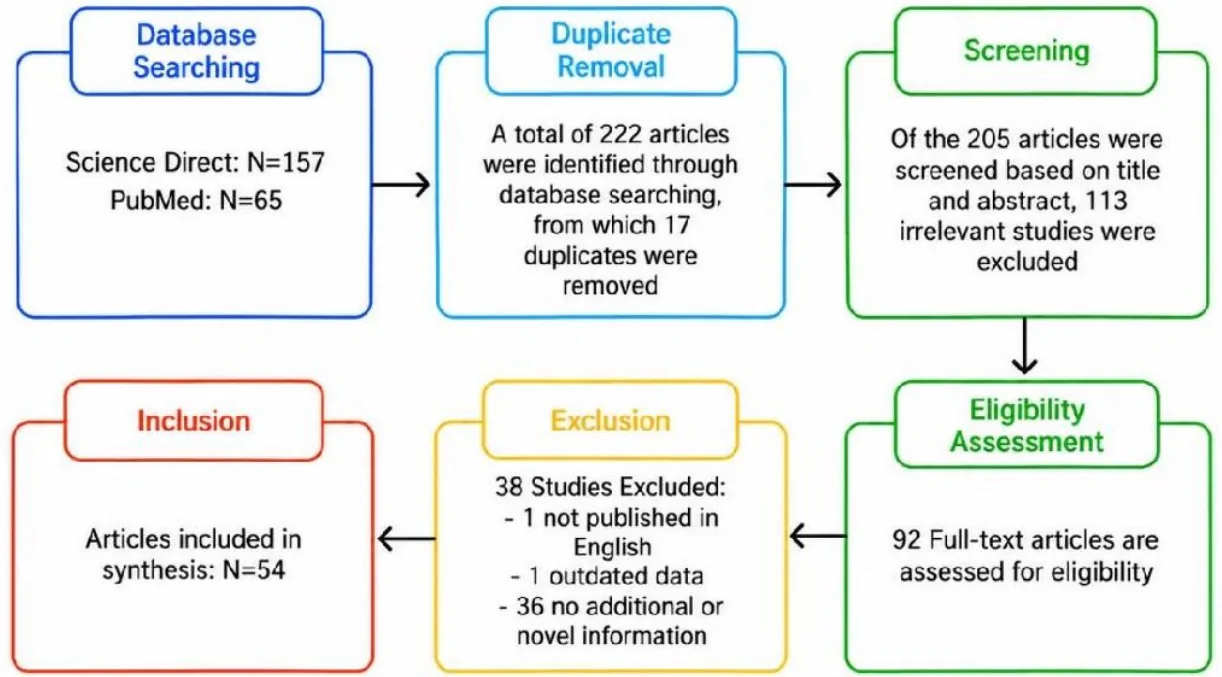
Flow diagram of the study selection process.

**Table 1:** Global epidemiological overview of candidemia [1,3].

Country	Incidence (per 100,000)	*C. albicans*/NCACs Ratio	Mortality (30 Days)
USA	13.3–26.2	37%/63%	28–29%
France	3.6	56%/44%	40%
Spain	8.1	45%/55%	31%
Iceland	5.7	56%/44%	30%
Brazil	12.5	34%/66%	54%

**Table 2:** Trends, geographical zones, and resistance of NCACs.

Species	Trends and Geographical Zones	Emerging Resistance
*C. glabrata*	Sharply increasing in the USA, Australia, and Northern Europe.	Growing resistance to azoles and echinocandins [8].
*C. parapsilosis*	Very common in Latin America, Asia, and Southern Europe.	Emergence of fluconazole-resistant strains (>50% in some series) [1].
*C. tropicalis*	Predominant in Asia and Latin America.	Fluconazole resistance ranges from 7% to 43% in some Asian countries [1].
*C. krusei*	Lower but stable incidence (~2–3%).	Intrinsic resistance to fluconazole [8].

**Table 3:** Phenotypic and Immunological Divergences among major Candida species.

Species	Primary Morphology	Size (µm)	Key Sensors (PRRs)	Dominant Cytokine Pathway	Evasion Strategy
*C. albicans*	Yeast, Pseudohypha, and Hypha	4–10	Dectin-1, Dectin-2, TLR2/4	IL-17 (Mucosae) and IFN-γ	β-glucans masking (Hyphae)
*C. glabrata*	Yeast only	1–4	TLR2, Dectin-2 (low Dectin-1)	IFN-γ, IL-12	Persistence and replication in macrophages
*C. tropicalis*	Yeast, Pseudohypha, and Hypha	5–11	Dectin-1, CARD9	TNF-α	Resistance to neutrophil killing
*C. parapsilosis*	Yeast and Pseudohypha,	3–9	TLR4, Dectin-1	IL-17/IL-1	Biofilms on catheters and low intrinsic virulence
*C. auris*	Yeast (rarely pseudohyphae)	2.5–5	Complement system (C5), TLR-4, Dectin-2	IFN-γ, IL-6, IL-17	Thermostability (42 °C) and resistance to C5-mediated lysis

**Table 4:** Synthesis of host–pathogen interactions—Protective immunity mechanisms and *Candida* evasion strategies [7,11,12,13,16,17,19,20].

Immune Axis	Protection Mechanisms (Controls)	Subversion Strategies (Barriers)
Recognition	Direct detection of β-glucans and mannans via Dectin-1/2 and TLR2/4 complexes [7,11]. Targeting of specific β-1,2 linkages in *C. auris* mannans by early IgM antibodies [13].	PAMP masking during hyphal transition, preventing immune alert [13]. High density of mannoproteins protecting internal targets and structural variability between strains [13].
Cellular Response	Coordinated Th1 (systemic) and Th17 (mucosal) polarization via CARD9/NLRP3 axis [11]. PD-1/PD-L1 blockade to reverse T-cell exhaustion [15].	Fungal-driven shift toward non-protective Th2 responses or expansion of immunosuppressive Tregs [11].
Morphology	Vaccines targeting adhesion proteins (Als3p) to block filamentation [7,11].	Morphological plasticity allows tissue penetration and physical breaching of epithelial barriers [7,11].
Localization	DC specialization (Langerhans cells for Th17) according to tissue depth [17].	Compartmentalization: local immunity (e.g., vaginal) does not guarantee systemic protection [11,17].
Complement System	C5 cascade activation essential to prevent systemic dissemination (A/J model) [14].	Intrinsic resistance of certain strains to opsonization in hosts with partial complement deficiencies [14].
Humoral/Soluble	Opsonophagocytosis; neutralization of SAPs and Hsp90; biofilm disruption; direct candidacidal activity [19]. Synthetic disaccharide 19 for rapid diagnosis and subunit vaccines [13].	Degradation of IgG/IgA by fungal proteases; release of eHsp90 causing macrophage pyroptosis [18,19].

**Table 5:** NDV-3A Phase II Clinical Outcomes in Recurrent Vulvovaginal Candidiasis [32].

Study Parameters	Clinical Analysis & Study Parameters	Placebo Group (*n* = 188 Overall)	NDV-3A Vaccine Group (rAls3p-N)
Global Cohort Analysis (Ages 18–55)	Overall RVVC Frequency Reduction	Baseline Reference	Positive trend (Full statistical significance not reached)
Target Subgroup Analysis (Women < 40 Years Old)	Median Time to First Symptomatic Episode	105 Days	210 Days (Doubled; *p* value trend significant)
	Recurrence-Free Rate at 12 Months	22%	42%
Safety, Reactogenicity & Tolerability	Adverse Events (AEs) Profile	Baseline Reference	Highly favorable; restricted to mild, transient local reactions at injection site
	Clinical Validation Status	—	First successful large-scale clinical validation of a fungal vaccine

**Table 6:** Synergistic Survival (with Amphotericin B) Impact in Disseminated Candidiasis [15].

Treatment	Clinical Survival Rate
Amphotericin B monotherapy	48%
Amphotericin B + Efungumab (anti-Hsp90 scFv) (Mechanism: Disruption of structural Hsp90 stress response pathways)	84%

**Table 7:** Inclusion and exclusion criteria.

Inclusion Criteria	Exclusion Criteria
Original articles and reviews in English, in peer-reviewed journals	Opinion pieces, editorials, letters to the editor, and conference abstracts
Published between 1 January 2015, and 31 December 2025	Studies that did not present original data on vaccines
studies addressing the development, pre-clinical trials, or clinical trials of *Candida* vaccines	Duplicate publications

## Data Availability

No new data were created or analyzed in this study. Data sharing is not applicable to this article.
